# Impact of e-cigarettes as cancer risk: A protocol for systematic review and meta-analysis

**DOI:** 10.1097/MD.0000000000032233

**Published:** 2023-01-06

**Authors:** Kleyton Santos de Medeiros, Beatriz Ferreira Pereira Pacheco, Paula Ermans de Oliveira, Ivna Letícia de Góis Nogueira, Vinícius Romeu Beserra Diógenes, Fernanda Gadelha Fernandes, Gabriela Cunha Fernandes, Edilmar de Moura Santos, Amália Cinthia Menseses do Rêgo, Irami Araújo-Filho

**Affiliations:** a Instituto de Ensino, Pesquisa e Inovação, Liga Contra o Câncer, Natal, RN, Brazil; b Postgraduate Program in Health Sciences, Federal University of Rio Grande do Norte, Natal, RN, Brazil; c Department of Surgery, Federal University of Rio Grande do Norte, Natal, RN, Brazil.

**Keywords:** e-cigarette use, e-cig use, vaping, cancer, tumors, neoplasms

## Abstract

The use of electronic cigarettes is one of the current public health problems on increasing alert, has been growing at an accelerating rate, and has become a public health emergency. Its importance is explained by the continuous growth and acceleration of oncological rates among all ages versus the absence of high-quality evidence, correlated to the use of nicotine derived products, being at their regular versions or the new ones. Available preclinical data indicate that activation of the sympathetic nervous system by nicotine inhaled from e-cigarettes may stimulate cancer development and growth by several mechanisms, which results can significantly reduce life’s quality. This systematic review and meta-analysis protocol aims to clarify the connection between the use of electronic cigarettes by adults over the age of 18 and the development of malignant neoplastic diseases.

**Method:**

The proposed systematic review and meta-analysis will be reported conforming to the preferred reporting items for systematic reviews and meta-analyses guidelines. Will include the following studies: case-control or cohort studies showing adults (18 years old age) using e-cigarettes. There will be no language or publication period restrictions. Articles published, but not peer-reviewed, will not be included in the review. Data will be entered in the Review Manager software (RevMan5.2.3). For dichotomous outcomes, we extracted or calculated the OR and 95% CI for each study. In case of heterogeneity (*I*²>50%), the random-effects model will be used to combine the studies to calculate the OR and 95% CI.

## 1. Introduction

Electronic devices for smoking pose a serious threat to global health, as the heating of the products contained in electronic cigarettes, in addition to the addictive power of nicotine, produces condensed carcinogenic hydrocarbons and toxic products to the human body. An example of this is the presence of nickel and ethylene oxide, substances related to the emergence of lung and sinus neoplasms, lymphomas, multiple myeloma, and leukemia.^[1]^

In addition, chronic use of nicotine or smoking has been recognized as one of the main risk factors for several clinical entities and disease of its own by mechanisms, as defined in the international statistical classification of diseases and related health problems: E-cigarette or Vaping product use-Associated Lung Injury, being marked by micro and macro metabolic disorders, capable of maintaining continuous inflammatory processes in tissue cells, especially lung cells, by the action of these heated chemical substances that make up flavored e-juices.^[1,2]^

Conceived and patented by Chinese pharmacist Hon Lik in 2003 ^[2]^, electronic cigarette (EC) is purchased over the Internet or sold directly to the consumer in many countries. Like a traditional cigarette, EC imitates experiences, and some have essences, and with that, they attract a high number of customers, mostly young people from 14 years old on average.^[2,3]^

The safety of EC nicotine delivery has not been scientifically demonstrated, and the risk of acquiring the disease is undetermined. There is no safety in the use of electronic cigarettes (ECs) as well as regulation in quality control.^[4]^

The absence of control promotes a variety of devices and therefore the concentration of nicotine in the other constituents is different. Thus, it is impossible to identify what is being inhaled by the user, and that’s just where the biggest problem is, as some changes can be observed after diagnosis to the updated system, a fact that makes early diagnosis of the problem by cancer difficult.^[2,4]^

Considering the regular and direct mechanisms of aggression, it is known that oncological outcomes are also explained by the activation of the sympathetic nervous system from the nicotine inhaled from electronic cigarettes, as pointed out in pre-clinical data that attest to the constant stimulation of development and growth of cancer by several mechanisms, which significantly compromise the quality of life. Added to this, some changes can only be observed after years of injury, a fact that makes it more difficult to establish an early diagnosis, increasing overall morbidity and mortality.^[4]^

Therefore, the use of electronic cigarettes is one of the current public health problems on increasing alert, since 3.6 million youth U.S people maintained regular use in 2020, according to data from the FDA (U.S. Food & Drug Administration).^[3]^ Given its popularity and easy access over the last 5 years, with the supposed prerogative that these cigarettes are less harmful to health than conventional cigarettes or that they can be therapeutic alternatives to replace cigarettes in their usual version, the objective is to elucidate the relationship of these electronic devices and their neoplastic potential in adults.

### 1.1. Review question

What are the neoplastic outcomes of the use of e-cigarettes by people over the age of 18?

### 1.2. Objectives

This systematic review and meta-analysis protocol aims to clarify the connection between the use of electronic cigarettes by adults over the age of 18 and the development of malignant neoplastic diseases.

## 2. Materials and methods

The proposed systematic review and meta-analysis will be reported conforming to the preferred reporting items for systematic reviews and meta-analyses guidelines.^[5]^ This protocol is registered with the International Prospective Register of Systematic Reviews (CRD42022295324).

### 2.1. Inclusion criteria

This systematic review will include the following studies: case-control or cohort studies showing adults (18 years old age) using e-cigarettes. There will be no language or publication period restrictions. Articles published, but not peer-reviewed, will not be included in the review.

### 2.2. The PECOT strategy

• Population/participants: adults (18 years old age);
• Exposure: use of e-cigarettes;
• Comparator/control: nonsmokers;
• Outcome: cancer;
• Types of studies: observational studies (cohort and case-control).

### 2.3. Types of patients

Participants of the studies will be adults over 18 years diagnosed with cancer and exposed to e-cigarettes. There will be no other age or gender restriction.

### 2.4. Types of interventions

Studies that described adults diagnosed with malignant neoplastic diseases and exposed to eletronic cigarettes, to evaluate the neoplastic consequences of using those devices, in comparison to nonsmokers.

### 2.5. Type of outcome measures

#### 2.5.1. Neoplasic outcomes.

The primary outcome to be evaluated will be the development of cancer. There are no additional outcomes to be evaluated.

### 2.6. Patient and public involvement

This work is a systematic review protocol; the research will be performed with a wide and comprehensive search of literature from databases, and individual patient data will not be included. Thus, the authors will not involve patients when setting the search questions and determining the outcome measurements during the design and implementation of the study, and in the dissemination of the results.

### 2.7. Search strategy

The studies will be obtained through PubMed, ScienceDirect, Embase, CINAHL, LILACS, CENTRAL, Web of Science, Scopus, Cochrane Library databases. “Grey literature” will be searched in www.opengrey.eu, without restrictions on the search for languages and year of publication. Articles will also be searched from the references of the selected studies, and the search strategy used in PubMed is shown in Table 1.

**Table 1:** Medline search strategy.

Search items	
1	Vaping
2	E-Cigarette use
3	E-Cisg use
4	ECig use
5	ECigarette use
6	Eletronic cigarette use
**7**	Nicotine vaping
8	Vape
9	Nonsmokers
10	Non-smokers
11	Nonsmokers
12	Neoplasmss
13	Neoplasms, malignant
14	Malignancy
15	Malignancies
16	Tumors
17	Cancer
18	Observational study
19	Cohort studies
20	Retrospective studies

EC = electronic cigarette.

The medical subject heading (MESH) terms will be: (Vaping OR E-Cigarette Use OR E-Cig Use OR ECig Use OR E Cigarette Use OR Eletronic Cigarette Use OR Nicotine Vaping OR Vape) AND (Nonsmokers OR Non-Smokers OR Nonsmoker) AND (Neoplasms OR Neoplasms, Malignant OR Malignancy OR Malignancies OR Tumors OR Cancer) AND (Observational Study OR Cohort Studies OR Retrospective Studies) (Table 1).

### 2.8. Other sources

Eligible studies can also be selected from the reference lists of retrieved articles. That is, the scope of the computerized literature search may be enlarged based on the reference lists of retrieved articles.

### 2.9. Data collection and analysis

#### 2.9.1. Selection of studies.

**Figure 1.**
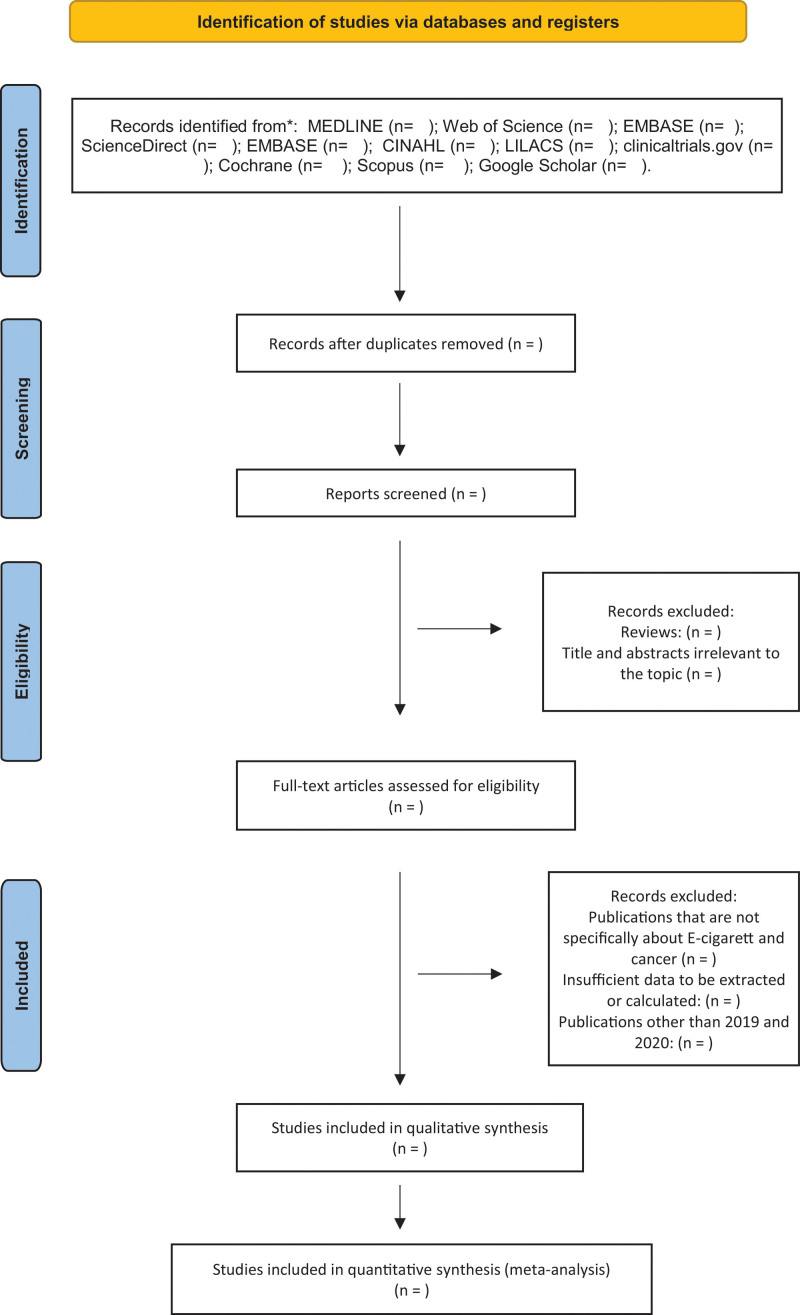
PRISMA flow diagram for systematic review and meta-analysis. PRISMA = preferred reporting items for systematic reviews and meta-analyses.

Two researchers (BFPP and VRBD) participated in the selection of the studies of interest using Ryan Software. Titles and abstracts will be read independently, and duplicate studies will be excluded. The same authors analyzed the selected texts in order to assess compliance with the inclusion criteria. A third reviewer, KSM, will solve the discrepancies. The selection of studies will be summarized in a preferred reporting items for systematic reviews and meta-analyses flow diagram (Fig. 1).

#### 2.9.2. Data extraction and management.

A standardized data extraction form will be developed and tested. Data from each included study will be extracted independently by 2 reviewers (PEO and ILGN), and any subsequent discrepancies will be resolved through discussion with a third reviewer (KSM). The data extracted will include information on authors, year of publication, study location, study design, data collection, number of cases, number of controls, follow up of participants and type of cancer. Furthermore, participant characteristics will be extraction (e.g., mean age, gender, ace/ethnicity).

#### 2.9.3. Addressing missing data.

In the case of missing data, the authors of this article will contact the corresponding authors or coauthors of the article, by phone or email. If we do not receive the necessary information, the data will be excluded from our analysis and will be covered in the discussion section.

#### 2.9.4. Risk of bias assessment.

Two authors, GCF and FGF, will independently assess the risk-of-bias in the eligible studies using the Newcastle-Ottawa scale for observational studies.^[6,7]^ Bias will be assessed as a judgement (high, low or unclear) for individual elements from 8 domains. A high score indicates a low risk of bias. Raters will resolve disagreements by consensus. A fourth rater will arbitrate cases for which consensus is unreachable.

#### 2.9.5. Assessment of heterogeneity.

We will use the *χ*^2^ test to evaluate the study outcomes (significance level of *P* < .1). The evaluation of the heterogeneity will be realized according to the Cochrane Handbook criteria through the *I*^2^ statistic. We consider that a value of 0% demonstrates a lack of heterogeneity in studies; ≥50% values indicate considerable heterogeneity. It is essential to mention that this evaluation will be executed if the meta-analysis’s achievement was appropriate.

If the *I*^2^ value is less than 50%, the heterogeneity is low, and a fixed-effect model will be used in the analysis. Otherwise, the heterogeneity will be considered high if the *I*^2^ value is 50% or more, and a random effects model will be used. Forest plots will be constructed to show the study-specific RR/OR estimates and pooled RR/OR estimates. Along with the forest plots, we will use Eggers’s test and Duval and Tweedie’s trim-and-fill method.

### 2.10. Analysis

Data will be entered in the Review Manager software (RevMan5.2.3) https://training.cochrane.org/online-learning/core-software/revman. This software allows the user to enter protocols; complete reviews; include text, characteristics of the studies, comparison tables, and study data; and perform meta-analyses. For dichotomous outcomes, we extracted or calculated the OR and 95% CI for each study. In case of heterogeneity (*I*² > 50%), the random-effects model will be used to combine the studies to calculate the OR and 95% CI, using the DerSimonian–Laird algorithm in the meta for the package, which provides functions for conducting meta-analyses in *R*.

### 2.11. Grading quality of evidence

To grade the strength of evidence from the included data, we will use the grading of recommendation assessment, development, and evaluation approach.^[8]^ The summary of the assessment will be incorporated into broader measurements to ensure the judgment of the risk of bias, consistency, directness, and precision. The quality of the evidence will be assessed based on the risk of bias, indirectness, inconsistency, imprecision, and publication bias. Tool classifies the studies as low, moderate and high quality. Two authors will independently make this evaluation, and any disagreements will be decided through discussion (third author).

## 3. Discussion

The increased use of e-cigarettes, in first place, initiated as a facilitation of smoking cessation, has brought to light the discussion of the relationship between its use with the neoplastic actual index promoted by this new modality of nicotine consume and, somehow, as a replacement habit that stimulate the activity of smoking.^[9]^ Nevertheless, as demonstrated by Hartmann-Boyce et al in a Cochrane review, this nicotine replacement is not essential for “Nicotine Replacement therapy” to be effective.^[10]^ As well, vaping devices when compared with tobacco have a retention rate of nicotine around 99%,^[11]^ showing a problem in this substitution, because that substance itself has potent potential carcinogenic activity.^[9]^

Additionally, despite all the thought of electronic cigarettes as a device that helps people quit smoking and the problem with nicotine use, present data suggest an increasingly worrying rise on all these nicotine and vaping products between younger people in the US.^[12]^ Furthermore, in this context arises the need to research about e-cigarettes and its oncological consequences in adults over 18 years. Otherwise, there are only a few studies that establish such an approach. As long as these studies emerge, despite the advertising selling e-cigarettes as less harmful and oncogenic than the other cigarettes with nicotine, they are far from benign. Consequently, this study aims to show a discussion of the ongoing investigation about the neoplastic outcome of electronic cigarettes.

According to a systematic review of the European Urology Oncology^[11]^ the damage caused by toxic substances that are in the e-cigarettes composition can directly impact the development of cancerigenous cells, mostly seen on the established role that its consume plays in the bladder cancer setup. Another finding from that study, is that the urine from electronic cigarettes users contains carcinogens that have a strong link to bladder cancer. Although the study claims that the malignant potential of e-cigarettes for that type of cancer remains unknown.

The cited systematic review only explores 1 type of malignancy, the urologic ones, especially bladder cancer, which makes clear the ultimate need of further papers to deepen the discussion and amplify our Acknowledgments on its subject. Another systematic review published at Preventive Medicine,^[2,4]^ shows experimental studies that have found effects after very short-term exposure to ECs which are reminiscent of the obstructive effects seen with smoking. As well, that exposure and the consequential effect of nicotine can impact cognitive and health performance, respiratory symptoms, and general health. It is known that the exposure to glycerol, a substance present on the ECs, irritates the upper respiratory tract and brings squamous metaplasia of the epiglottis.^[2]^ In general, harmful substances detected on electronic cigarettes are at low concentrations, but can cause damage with intense and chronic exposure.^[3]^

Currently, the expectation is to see the cancer outcomes later than acute respiratory and cardiovascular events since these effects have a lengthy induction time.^[12]^ Especially because the smoking-related risk with neoplasic outcomes need to be observed and have a population who had smoked for long enough for the effects to become fully manifest, as it was with the relation of tobacco and lung cancer,^[13,14]^ but all of the studies cited at the present discussion did not have time yet to observe the malignant transformation. The direct, mammalian oncogenicity of electronic cigarette smoke (ECS) – delivered nicotine and its nitrosamine products was demonstrated in a murine model by Tang et al In this study were exposed respectively over a 54-week period to either aerosolised e-liquid; the, apparently, inert organic vehicle or filtered air. A lung cancer incidence was 22.5 % in the E-cigarette arm versus 5.6 % and 0% in the filtered air and vehicle arms, respectively – with this result achieving significance, bringing an idea of caution and prudence with vaping.^[9,14–16]^

## 4. Ethics and dissemination

Ethical approval is not required because this review will draw on publicly available scientific literature. Findings of this systematic review will be published in a peer-reviewed journal and updates will be conducted if there is enough new evidence that may cause any changes in the conclusions of the review. Any amendments made to the protocol during the conduct of the review will be reported in the manuscript.

## Acknowledgments

The authors acknowledge the assistance provided by the Graduate Program in Health Sciences of the Federal University of Rio Grande do Norte (UFRN) in the undertaking of literary research, and teaching, research and innovation institute of Liga Contra o Câncer, Natal-RN, Brazil.

## Author contributions

**Conceptualization:** Kleyton Santos de Medeiros, Beatriz Ferreira Pereira Pacheco, Paula Ermans Oliveira, Ivna Letícia Góis Nogueira, Vinícius Romeu Beserra, Fernanda Gadelha Fernandes, Gabriela Cunha Fernandes, Edilmar de Moura Santos, Amália Cinthia Menseses do Rêgo, Irami Araújo-Filho.

**Investigation:** Kleyton Santos de Medeiros, Gabriela Cunha Fernandes.

**Methodology:** Kleyton Santos de Medeiros.

**Project administration:** Kleyton Santos de Medeiros, Edilmar de Moura Santos, Irami Araújo-Filho.

**Resources:** Beatriz Ferreira Pereira Pacheco.

**Supervision:** Kleyton Santos de Medeiros, Irami Araújo-Filho.

**Validation:** Kleyton Santos de Medeiros, Edilmar de Moura Santos, Amália Cinthia Menseses do Rêgo, Irami Araújo-Filho.

**Visualization:** Kleyton Santos de Medeiros, Paula Ermans Oliveira, Ivna Letícia Góis Nogueira, Vinícius Romeu Beserra, Fernanda Gadelha Fernandes, Gabriela Cunha Fernandes, Edilmar de Moura Santos, Amália Cinthia Menseses do Rêgo, Irami Araújo-Filho.

**Writing – original draft:** Kleyton Santos de Medeiros, Beatriz Ferreira Pereira Pacheco, Paula Ermans Oliveira, Ivna Letícia Góis Nogueira, Vinícius Romeu Beserra, Fernanda Gadelha Fernandes, Gabriela Cunha Fernandes, Irami Araújo-Filho.

**Writing – review & editing:** Kleyton Santos de Medeiros, Edilmar de Moura Santos, Amália Cinthia Menseses do Rêgo, Irami Araújo-Filho.
